# Understanding the Impact of Menopause on Women With Schizophrenia-Spectrum Disorders: A Comprehensive Review

**DOI:** 10.7759/cureus.37979

**Published:** 2023-04-22

**Authors:** Swasti Tiwari, Roshan Prasad, Mayur B Wanjari, Ranjana Sharma

**Affiliations:** 1 Community Medicine, Jawaharlal Nehru Medical College, Datta Meghe Institute of Higher Education and Research, Wardha, IND; 2 Medicine and Surgery, Jawaharlal Nehru Medical College, Datta Meghe Institute of Higher Education and Research, Wardha, IND; 3 Research and Development, Jawaharlal Nehru Medical College, Datta Meghe Institute of Higher Education and Research, Wardha, IND; 4 Medical Surgical Nursing, Smt. Radhikabai Meghe Memorial College of Nursing, Datta Meghe Institute of Higher Education and Research, Wardha, IND

**Keywords:** biological changes, women health, schizophrenia spectrum disorders, schizophrenia, menopause

## Abstract

Menopause is a physiological event in women's lives that typically transpires in midlife, denoting the cessation of ovarian function and ultimately leading to the end of reproductive capacity. However, women with schizophrenia-spectrum disorders may encounter unique challenges during this period because of the interaction between hormonal changes and their pre-existing mental health conditions. This literature review aims to investigate the consequences of menopause on women with schizophrenia-spectrum disorders, including modifications in symptomatology, cognitive function, and quality of life. Potential interventions will also be examined, including hormone replacement therapy and psychosocial support. The study findings suggest that menopause can worsen symptoms, such as hallucinations and delusions, and may also impair cognitive function, resulting in memory and executive function difficulties. Nevertheless, hormone replacement therapy and psychosocial support could offer potential avenues to manage symptoms and improve the quality of life for women with schizophrenia-spectrum disorders during menopause.

## Introduction and background

Menopause is a physiological process characterized by the permanent cessation of menstrual periods, marking the end of a woman's reproductive capacity, which typically occurs between the ages of 45 and 55 years [1]. This process involves a decrease in ovarian function, which reduces estrogen production and a variety of physical and psychological symptoms [2]. On the other hand, schizophrenia-spectrum disorders are severe and chronic mental illnesses that affect an individual's thoughts, emotions, and behavior. While menopause and schizophrenia-spectrum disorders are distinct entities, they frequently co-occur in women. According to the fifth edition of the Diagnostic and Statistical Manual of Mental Disorders, the lifetime prevalence of schizophrenia appears to be approximately 0.3-0.7%, although there is reported variation by race/ethnicity, across countries, and by geographic origin for immigrants and children of immigrants [3]. During their reproductive years, women have a higher prevalence of schizophrenia-spectrum disorders, and those with schizophrenia are more likely to experience early menopause than the general population [4,5]. Thus, a comprehensive review of the existing literature is necessary to provide insight into the effects of menopause on the course and outcome of schizophrenia-spectrum disorders in women.

The interaction of menopause and schizophrenia-spectrum disorders is a relatively under-researched area, with limited knowledge about the impact of menopause on the course and outcome of schizophrenia-spectrum disorders in women. While some studies suggest that menopause may exacerbate symptoms of schizophrenia-spectrum disorders [6,7], others have found no significant association. Furthermore, there is no agreement on the optimal management of menopausal symptoms in women with schizophrenia-spectrum disorders, which can complicate these patients' overall treatment and care [8,9]. The lack of consensus regarding the effect of menopause on schizophrenia-spectrum disorders in women highlights the need for further research in this area.

This review aims to provide a thorough overview of how menopause affects women with schizophrenia-spectrum disorders. The article will cover key areas, such as an introduction to menopause and schizophrenia-spectrum disorders, including the prevalence of these conditions. Additionally, it will summarize the biological changes that occur during menopause and their implications for women with schizophrenia-spectrum disorders. Furthermore, it will offer a critical evaluation of the impact of menopause on symptoms of schizophrenia-spectrum disorders, including positive, negative, and cognitive symptoms. The review will also include an analysis of the impact of menopause on psychosocial functioning, including social functioning, quality of life, and family relationships. Lastly, the article will provide an overview of the treatment and management considerations for menopausal symptoms in women with schizophrenia-spectrum disorders.

## Review

Methodology

We undertook a systematic approach to conduct a thorough literature review on the impact of menopause on women with schizophrenia-spectrum disorders. We searched relevant electronic databases like PubMed, PsycINFO, and Google Scholar and manually searched relevant journals and reference lists to identify pertinent articles. Our search strategy comprised a combination of keywords and medical subject headings (MeSH) terms related to women's health, menopause, and schizophrenia-spectrum disorders. To be eligible for inclusion in the review, studies had to focus on menopause and its impact on women with schizophrenia-spectrum disorders and be published in English.

To ensure the quality of our review, two reviewers, RP and MW, independently screened the titles and abstracts of the identified articles for relevance. The full text of potentially eligible articles was reviewed, and one reviewer extracted the relevant data, which another reviewer then cross-checked. We organized the review into sections based on the main themes from the literature, including the physiological changes during menopause, the impact on symptoms of schizophrenia-spectrum disorders, and the psychosocial and treatment considerations. We synthesized the data from each section and presented it in a narrative format to present the findings.

Our review provides a comprehensive and up-to-date understanding of the impact of menopause on women with schizophrenia-spectrum disorders, highlighting important considerations for both healthcare providers and patients. Overall, this review serves as a valuable resource for healthcare professionals and researchers who are interested in understanding the impact of menopause on women with schizophrenia-spectrum disorders.

Menopause and schizophrenia-spectrum disorders: an overview

Menopause and schizophrenia-spectrum disorders are distinct yet interconnected conditions that frequently co-occur in women. As estrogens act as a protective factor in women regarding the onset of schizophrenia, their increase in puberty helps delay the onset of symptoms. At the same time, their abrupt decline in menopause may account for a second peak of onset and greater severity of the symptoms. Genetic predisposition, medication use, lifestyle factors, and social determinants of health may contribute to this heightened risk [10-13].

The interaction between menopause and schizophrenia-spectrum disorders is complex and multifaceted. Women with schizophrenia-spectrum disorders may experience more severe and frequent menopausal symptoms like increased mood swings and altered sleep than the general population, possibly due to the physiological and psychological stress associated with their condition. Additionally, menopause may worsen symptoms of schizophrenia-spectrum disorders, including mood disturbances, cognitive deficits, and positive and negative symptoms [14-17].

Biological changes during menopause

Menopause is a physiological process. The hormonal changes during menopause are intricate and profoundly impact various physiological systems in the body, such as the reproductive, cardiovascular, skeletal, and central nervous systems [18,19]. Estrogen, a crucial hormone, plays a significant role in a woman's reproductive and overall health. It regulates the growth and development of reproductive tissues, controls the menstrual cycle, and supports bone health. Furthermore, estrogen significantly impacts the brain and affects many cognitive and affective processes, including learning and memory, mood regulation, and stress response [20,21].

During menopause, the decline in estrogen production can lead to various physiological changes in the body, resulting in several physical and psychological symptoms, including hot flashes, night sweats, vaginal dryness, mood changes, and cognitive deficits [22]. In addition to these symptomatic changes, menopause has significant long-term implications for women's health. One of the most significant long-term effects of menopause is the increased risk of osteoporosis, which results in decreased bone density and an increased fracture risk [23]. Estrogen plays a critical role in bone health by inhibiting bone resorption and promoting bone formation [24]. Therefore, the decline in estrogen levels during menopause leads to increased bone turnover and decreased bone density, which can result in osteoporosis and related fractures [25].

The decline in estrogen levels during menopause also impacts cardiovascular health. Estrogen protects the cardiovascular system by promoting vasodilation, reducing inflammation, and lowering low-density lipoprotein cholesterol levels. Therefore, decreased estrogen production during menopause increases the risk of cardiovascular diseases, including coronary heart disease and stroke [26,27]. Lastly, menopause affects the central nervous system, particularly the brain. Estrogen has a neuroprotective effect and is involved in various cognitive processes, such as memory, attention, and executive function. Consequently, the decline in estrogen production during menopause can lead to cognitive deficits and an increased risk of neurodegenerative diseases, such as Alzheimer's [25,27].

Impact of menopause on symptoms of schizophrenia-spectrum disorders

Menopause could potentially exacerbate schizophrenia-spectrum disorders, such as mood disturbances, cognitive deficits, and positive and negative symptoms. For instance, Seeman conducted a study that showed that postmenopausal women with schizophrenia had significantly lower verbal memory test scores compared to premenopausal women with schizophrenia. Similarly, another study revealed that postmenopausal women with schizophrenia had significantly lower scores in tests measuring verbal fluency, attention, and working memory than premenopausal women with schizophrenia. These findings suggest menopause could exacerbate cognitive deficits in women with schizophrenia-spectrum disorders [28-31].

Besides cognitive deficits, menopause could worsen mood disturbances in women with schizophrenia-spectrum disorders. A study conducted by El khoudary et al. found that postmenopausal women with schizophrenia had higher levels of depression and anxiety than premenopausal women with schizophrenia [32]. Another study showed that postmenopausal women with schizophrenia had higher negative symptoms, including apathy and social withdrawal, than premenopausal women with schizophrenia [33]. These findings suggest menopause could worsen mood disturbances and negative symptoms in women with schizophrenia-spectrum disorders. However, the effect of menopause on positive symptoms, such as hallucinations and delusions, is not as clear. Some studies have not found any significant association between menopause and positive symptoms in women with schizophrenia-spectrum disorders [34,35]. Other studies have suggested that menopause could worsen positive symptoms [16,36]. Further research is necessary to clarify the impact of menopause on positive symptoms in women with schizophrenia-spectrum disorders.

Impact of menopause on psychosocial functioning

Menopause, with its physical and cognitive changes, significantly impacts psychosocial functioning, leading to emotional and social challenges for women. Hormonal changes during menopause can result in mood disturbances such as anxiety and depression, negatively impacting psychosocial functioning [37]. Additionally, symptoms like hot flashes and night sweats can disrupt sleep, leading to fatigue and impacting emotional well-being and social functioning [38]. In addition to psychological effects, menopause can also affect sexual functioning and satisfaction, leading to a decline in psychosocial functioning and overall quality of life [39]. Decreased estrogen production, vaginal dryness, and atrophy can cause discomfort during intercourse and reduce sexual desire [40].

Furthermore, menopause can bring about a shift in social roles and relationships, influencing psychosocial functioning. Women experience changes in their relationships with their partners, children, and friends, leading to isolation, loneliness, and loss in their relationships [41]. Despite the challenges, menopause can be a time for women to grow and discover themselves. Many women report feeling more confident and empowered after menopause and take this opportunity to explore new interests and relationships [38].

Treatment and management considerations

Managing menopause-related symptoms in women with schizophrenia-spectrum disorders poses a significant challenge, as the available treatment options for menopause can potentially interact with psychiatric medications [42]. Therefore, a comprehensive approach that includes pharmacological and non-pharmacological interventions is necessary. Although hormone therapy (HT) is the most effective treatment for menopausal symptoms, like hot flashes, vaginal dryness, and mood changes [43], its use in women with schizophrenia-spectrum disorders remains controversial due to the potential exacerbation of psychiatric symptoms by estrogen [44]. Additionally, some antipsychotic medications used to manage schizophrenia-spectrum disorders can interact with HT, leading to adverse effects [45].

Non-pharmacological interventions, including cognitive-behavioral therapy (CBT), relaxation techniques, and physical exercise, may be useful in managing menopausal symptoms in women with schizophrenia-spectrum disorders [46]. CBT can help manage hot flashes and mood disturbances, while relaxation techniques, such as deep breathing and meditation, can assist in managing stress and anxiety. Physical exercise is also beneficial for managing hot flashes, enhancing mood, and promoting bone health [47]. In addition to managing menopausal symptoms, regular monitoring of bone health, cardiovascular health, and cognitive function is essential for women with schizophrenia-spectrum disorders. This can include routine bone density scans, lipid profile monitoring, and cognitive testing to identify changes or declines [48]. Table 1 summarizes the conclusion of various studies on the impact of menopause on the three primary domains of development of humans throughout their lifespan.

**Table 1:** Impact of menopause on the three primary domains of development of humans throughout their lifespan The author has recreated from the source [29,32,33,38,41].

Impact of menopause on	Result
Cognitive functioning	A decrease in verbal fluency, attention, and working memory.
Physical functioning	An increase in mood disturbances, sleep disturbances, irritation, decreased concentration, and hot flashes. A decreased sexual activity and satisfaction.
Psychosocial functioning	Increased levels of depression and anxiety. In addition, women experience changes in their relationships with their partners, children, and friends, leading to isolation and loneliness, which lead to apathy and social withdrawal.

## Conclusions

In conclusion, menopause can have a significant impact on women with schizophrenia-spectrum disorders, leading to changes in symptomatology, cognitive function, and quality of life. The interplay between hormonal changes and the existing mental health condition can pose unique challenges for these women. Although there is limited research on this topic, the existing evidence suggests that menopause can exacerbate symptoms of schizophrenia-spectrum disorders and may lead to cognitive difficulties. However, hormone replacement therapy and psychosocial support can be potential interventions for managing these symptoms and improving the quality of life for women with schizophrenia-spectrum disorders during menopause. It is crucial for healthcare providers to recognize the potential impact of menopause on women with schizophrenia-spectrum disorders and provide appropriate care and support. Future research in this area is necessary to provide a more comprehensive understanding of the impact of menopause on the course and outcome of schizophrenia-spectrum disorders in women. Ultimately, a better understanding of this interaction can lead to the development of more effective interventions and treatments for women with schizophrenia-spectrum disorders during menopause.
